# Distinct Effects of a High Fat Diet on Bone in Skeletally Mature and Developing Male C57BL/6J Mice

**DOI:** 10.3390/nu13051666

**Published:** 2021-05-14

**Authors:** Dean S. Ross, Tzu-Hsuan Yeh, Shalinie King, Julia Mathers, Mark S. Rybchyn, Elysia Neist, Melissa Cameron, Alexander Tacey, Christian M. Girgis, Itamar Levinger, Rebecca S. Mason, Tara C. Brennan-Speranza

**Affiliations:** 1 Faculty of Medicine and Health, School of Medical Sciences, University of Sydney, Sydney 2006, Australia; dros8694@uni.sydney.edu.au (D.S.R.); cindy.th.yeh@gmail.com (T.-H.Y.); shalinie.king@sydney.edu.au (S.K.); juliamathers94@gmail.com (J.M.); m.rybchyn@unsw.edu.au (M.S.R.); ellyn@hotmail.com (E.N.); melissa.cameron@sydney.edu.au (M.C.); rebecca.mason@sydney.edu.au (R.S.M.); 2 Faculty of Medicine and Health, School of Dentistry, University of Sydney, Sydney 2006, Australia; 3 Institute for Health and Sport (IHES), Victoria University, Melbourne 3011, Australia; alexander.tacey@live.vu.edu.au (A.T.); itamar.levinger@vu.edu.au (I.L.); 4 Australian Institute for Musculoskeletal Science (AIMSS), University of Melbourne and Western Health, St Albans 3021, Australia; 5 Department of Diabetes and Endocrinology, Westmead Hospital, Sydney 2145, Australia; christian.girgis@sydney.edu.au; 6 Department of Endocrinology, Royal North Shore Hospital, Sydney 2065, Australia; 7 Faculty of Medicine and Health, University of Sydney, Sydney 2006, Australia; 8 Faculty of Medicine and Health, School of Public Health, University of Sydney, Sydney 2006, Australia

**Keywords:** high fat diet, preclinical, bone histomorphometry, osteoblasts, osteocytes, metabolism

## Abstract

Increased risks of skeletal fractures are common in patients with impaired glucose handling and type 2 diabetes mellitus (T2DM). The pathogenesis of skeletal fragility in these patients remains ill-defined as patients present with normal to high bone mineral density. With increasing cases of glucose intolerance and T2DM it is imperative that we develop an accurate rodent model for further investigation. We hypothesized that a high fat diet (60%) administered to developing male C57BL/6J mice that had not reached skeletal maturity would over represent bone microarchitectural implications, and that skeletally mature mice would better represent adult-onset glucose intolerance and the pre-diabetes phenotype. Two groups of developing (8 week) and mature (12 week) male C57BL/6J mice were placed onto either a normal chow (NC) or high fat diet (HFD) for 10 weeks. Oral glucose tolerance tests were performed throughout the study period. Long bones were excised and analysed for ex vivo biomechanical testing, micro-computed tomography, 2D histomorphometry and gene/protein expression analyses. The HFD increased fasting blood glucose and significantly reduced glucose tolerance in both age groups by week 7 of the diets. The HFD reduced biomechanical strength, both cortical and trabecular indices in the developing mice, but only affected cortical outcomes in the mature mice. Similar results were reflected in the 2D histomorphometry. Tibial gene expression revealed decreased bone formation in the HFD mice of both age groups, i.e., decreased osteocalcin expression and increased sclerostin RNA expression. In the mature mice only, while the HFD led to a non-significant reduction in runt-related transcription factor 2 (Runx2) RNA expression, this decrease became significant at the protein level in the femora. Our mature HFD mouse model more accurately represents late-onset impaired glucose tolerance/pre-T2DM cases in humans and can be used to uncover potential insights into reduced bone formation as a mechanism of skeletal fragility in these patients.

## 1. Introduction

It is well known that patients with type 2 diabetes mellitus (T2DM), insulin resistance and obesity are at high risk for developing cardiovascular complications and retinopathy. Recent evidence has indicated that these patients are also at risk of increased falls and fractures [1,2,3,4,5,6], leading to reduced quality of life and increased risk of premature mortality [7]. The increased fracture risk in these patients may be unexpected, as patients with insulin resistance, T2DM and/or obesity have normal-to-high bone mineral density (BMD) [7,8,9,10], which is usually associated with higher bone quality and reduced fracture risk. Despite the increased BMD in these patients, the fracture risk is up to 3-fold higher compared to the general population [7,9,10,11]. This increase in fractures is independent of factors such as age, sex, body mass index, tendency to fall and visual impairment [8,10], indicating that this increased fracture risk is driven by compromised bone quality in these patients. As obesity, impaired glucose tolerance and T2DM have become a public health crisis of epidemic proportions [12], it is important to understand the mechanisms behind the skeletal fragility observed in patients with impaired glucose regulation. The health, social and economic impacts of fractures create extraordinary burdens that may be eased with the correct therapeutic intervention(s).

Evidence, in humans, currently indicates decreased bone quality associated with modifications to bone turnover, bone mineral quality and bone microarchitecture, although the exact mechanism underlying bone fragility in patients with T2DM is yet to be defined. It is therefore important to develop animal models that can be used to determine the structural, cellular and molecular mechanisms behind these skeletal changes. Patients with T2DM have reduced markers of bone remodelling [13]: procollagen type 1 N-terminal propeptide [14,15], osteocalcin [14,15,16], alkaline phosphatase [17] and carboxy-terminal cross-linked telopeptide of type I collagen [15,16,17], indicative of decreased bone turnover rate. In post-menopausal osteoporosis, high bone turnover results in low BMD, implying traditional anti-osteoporotic medications that aim to reduce bone turnover are not useful in patients with T2DM. Additionally, levels of sclerostin, a known Wnt antagonist and potent bone formation inhibitor secreted by osteocytes, is increased in patients with T2DM [17,18]. Another potential causal pathway in the skeletal fragility observed in these patients is the increased accumulation of advanced glycation endproducts (AGEs) [19,20]. Increased levels of AGEs, as seen in patients with T2DM [21], are likely due to the increased level of extracellular glucose and oxidative stress associated with the disease. AGEs adversely affect the mechanical properties of bone, and increase fracture rates [22,23]. Assessments of bone microarchitecture in patients with T2DM have had inconsistent outcomes. Decreased trabecular bone scores have been reported in patients with insulin resistance and T2DM [24,25]. However, most studies assessing trabecular microarchitecture via high resolution quantitative computed tomography (HR-QCT) and micro-computed tomography (µCT) have reported no changes [14,15,26,27], or improved trabecular bone volume to total volume ratio (BV/TV) [28,29,30]. Alternatively, some studies have reported altered cortical bone parameters in patients with glucose intolerance and T2DM, including the following: decreased cortical vBMD [27]; increased cortical porosity [27,28]; and increased cortical pore volume [28,31].

Rodent studies that have been used to investigate the mechanism behind reduced bone quality in cases of impaired glucose homeostasis or insulin resistance are yet to produce a phenotype that accurately reflects outcomes in humans, particularly relating to BMD and bone microarchitecture. This is likely due to previous studies placing little focus on the ‘time of onset’ when inducing the T2DM phenotype, and therefore investigating outcomes in mice that are still skeletally immature [32,33]. These models are therefore replicating a T1DM-like phenotype in which the reduction in bone size and skeletal pathology is considerably more marked [10] and easy to detect via dual-energy X-ray absorptiometry (DXA).

We aimed to develop this HFD-induced impaired glucose tolerance-like phenotype in mice, in which skeletal outcomes closely mimic those in humans with adult-onset early stage T2DM. We hypothesized that the T2DM-like phenotype will have more severely compromised functional and morphological skeletal outcomes when the age of onset of the HFD is prior to the acquisition of peak bone mass [34] compared to skeletally mature mice. This observation would result in the identification of a skeletal phenotype in the mature mice that closely mimics the observed bone geometry in adult-onset glucose intolerance, with normal to high BMD, yet increased fracture risk. 

## 2. Methods

### 2.1. Animals and Tissue Sampling

Male C57BL/6J mice were purchased from the Animal Resources Centre (Perth) at 6 weeks and 10 weeks of age and were then acclimatized for two weeks. Animals were maintained in individually ventilated cages, with up to 4 mice per cage in a light environment and alternating 12/12 h light/dark cycles. The temperature was controlled at 22 °C with 50% humidity at the University of Sydney Laboratory Animal Services in the physical containment 2 (PC2) animal facility. Mice had *ad libitum* access to water and food except when fasting for experiments. Mice were randomly assigned (*n* = 7 per group) either a 60% high fat diet (SF02-006), or continued on the normal chow (NC) diet (SF09-091) purchased from Specialty Feeds (Perth), with 8 week old mice representing the developing young adult group, and 12 week old mice representing skeletally mature adult groups [34]. The digestible energy content of the normal chow diet was 16.1 MJ/kg and that of the high fat diet was 24 MJ/kg. The fat content of each diet was as follows: canola oil 70 g/kg for normal chow; canola oil 100 g/kg + cocoa butter 400 g/kg + clarified butter 100 g/kg for the high fat diet.

Diets were maintained for 10 weeks after which the mice were euthanized by cardiac exsanguination under gaseous isoflurane anesthesia, followed immediately by decapitation. 

Bones were surgically excised for an ex vivo analysis. The left tibiae were removed and cleaned of soft tissue before being fixed in paraformaldehyde for 12 h and subsequently stored in 70% ethanol for µCT scanning. This was followed by decalcification and tissue processing for histology. The right tibiae were thoroughly cleaned of any soft tissue and flushed of all bone marrow before being snap frozen in liquid nitrogen and stored at −80 °C until RNA extraction was performed. The right femora were snap frozen and kept at −80 °C prior to protein extraction for the Western blot analysis. The left femora were excised immediately after sacrifice and frozen at −20 °C wrapped in saline soaked gauze in plastic bags. All animal procedures were approved by the University of Sydney Animal Ethics Committee, protocol number: 857, in line with the NSW Animal Research Act 1985 and the Australian code for the care and use of animals for scientific purposes 8th edition (2013).

### 2.2. Oral Glucose Tolerance Tests

Oral glucose tolerance tests (oGTT) were performed as previously described [35]. Briefly, mice were fasted for 6 h prior to performing the test. Baseline blood glucose readings were collected via tail vein prick and analysed using an Accu-Chek Performa II blood glucose monitor. An oral dose of glucose (2 g/kg total body weight) was then administered via gavage. Subsequent blood samples were measured as above at 15, 30, 60, 90 and 120 min after receiving the glucose dose.

### 2.3. Bone Microarchitecture by µCT

The tibia samples were wrapped in Parafilm^®^ M (Amcore) and scanned in air using the Bruker Skyscan 1172. Scans were performed with a voxel size of approximately 11 µm. X-ray source voltage and current were set to 100 kVp and 100 µA, respectively, with an exposure time of 590 ms through a 0.5 mm aluminum and copper filter. Scans were reconstructed using NRecon (version 1.6.9.18) with consistent parameters for all groups. Reconstructed scans were then orientated, and samples scanned together were separated using Dataviewer (64 bit V1.5.2.4). Next, CTan (64 bit V1.16) was used to define relative regions of interest, delineate cortical and trabecular bone by manually tracing and to analyze 3D parameters. 

### 2.4. Bone Histomorphometry 

Tibiae were decalcified in 10% ethylenediaminetetraacetic acid (pH 7), dehydrated in ethanol and embedded in paraffin for coronal sectioning at 5 µm thickness. Sections were stained with haematoxylin and eosin. A 4.5 mm^2^ region with an offset of 0.5 mm below the proximal growth plate was analysed in the 4 most medial sections of each mouse tibia. Trabecular bone parameters were calculated from bone traced manually at 400× magnification using OsteoMeasure Software (V4.2.0.0). Cell parameters were also measured manually using OsteoMeasure by defining osteoblasts cells as the polarized cells with cuboidal morphology at the bone surface [36], and osteocytes as cells fully encased in the bone matrix [37]. All histomorphometry was performed with the operator blinded to sample identification.

### 2.5. Biomechanical Bone Properties

Left femoral bones were slowly thawed at 4 °C overnight and then warmed to room temperature prior to mechanical testing. Bones were cleaned of soft tissue and the length and diameter were measured using digital calipers. The midline of the shaft was determined, and a line was drawn using a marker at this point. The bones were placed on the servo-controlled electromechanical system (Instron 1114, InstronCorp., High Wycombe, UK), on the horizontal plane, by laying the midshaft on two supports separated by 10 mm. A load was applied on the middle of the shaft as determined earlier, so as to achieve a three-point bending test. The actuator was displaced at 1 mm/min, and from the force–displacement curves we obtained the maximal load (N) and stiffness (slope of the linear part of the curve, representing the elastic deformation in N/mm) of the midshaft (a cortical-rich zone).

### 2.6. Real-Time Quantitative PCR

DNAse-treated total RNA was extracted from mouse tibiae, using TRIZOL reagent (Ambion by Life Technologies, Carlsbad, CA, USA) and QIAGEN RNeasy mini-kits. RNA was converted to cDNA using the Tetro cDNA synthesis kit (Bioline, Memphis, TN, USA), and amplification was performed on a Corbett Rotor Gene 6000 (QIAGEN, Germantown, MD, USA) with TaqMan Fast Universal PCR Master mix (Applied Biosystems by Thermo Fisher Scientific, Waltham, MA, USA) to quantify the presence of osteocalcin (Ocn), sclerostin (Sost) and runt-related transcription factor *2* (Runx2). This was completed using the following TaqMan Gene Expression Assay probes (Applied Biosystems by Thermo Fisher Scientific, Waltham, MA, USA): BGLAP Mm03413826_mH, SOST Mm00470479_m1 and Runx2 Mm00501584_m1, respectively. Furthermore, Gapdh Mm99999915_g1 was used as an internal reference. Real-time quantitative PCR was used for the determination of fold changes of gene expression. 

### 2.7. Western Blotting

The total protein was isolated from femora flushed on bone marrow using a combination of de-mineralization and chemical denaturation as previously described [38]. Protein extracts were precipitated from their chemical denaturation solutions and equal amounts of precipitate were used for Western blot analyses.

Precipitated protein was dissolved into 200 μL of 4 × Laemmli buffer (8% sodium dodecyl sulphate (SDS), 40% glycerol, 0.02% bromophenol blue, 250 mM Tris-HCl) by heating at 95 °C for 30 min. Next, 10 µL of β-mercaptoethanol was added and heated at 95 °C for an additional 15 min. Tubes were spun at 10,000× *g* at 25 °C for 5 min, and 10 µL supernatant was loaded into wells of 12% (*w*/*v*) tris-glycine polyacrylamide gel along with a BenchMark Pre-Stained molecular mass protein ladder. Protein was separated by electrophoresis at 100 V for approximately 60 min. The proteins were transferred onto a nitrocellulose membrane at 125 V for 75 min with the use of ice and an insulated container to prevent overheating.

Membranes were removed and washed 3 times in PBS + 0.002% tween. This was repeated a further 3 times after treatment with a blocking buffer (5% (*w*/*v*) bovine serum albumin in PBS) for 60 min to reduce non-specific binding. The membrane was then incubated overnight at 4 °C with Runx2 primary antibody (rabbit polyclonal, sc-10758; sc-47778; Santa Cruz Biotechnology, Dallas, TX, USA) at 1 μg/mL in the blocking buffer. The membrane was then treated with 0.1 μg/mL anti-rabbit Ig-HRP conjugated antibody (sc-2030; Santa Cruz Biotechnology) in the blocking buffer at room temperature for 1 h, and β-actin (sc-47778; Santa Cruz Biotechnology) was used as a loading control. The bands were developed using a chemiluminescent substrate. Image acquisition was achieved via a BioRad ChemiDoc imaging station. Densitometry detection was determined using Image J.

### 2.8. Statistical Analysis 

Statistical significance was determined between the experimental groups for µCT, bone histomorphometry and gene analysis by conducting a two-way analysis of variance (ANOVA) with a Bonferroni post-hoc analysis using GraphPad Prism 7 software. Response curves for the oGTTs were analysed using a general liner mixed model repeated measures analysis followed by Bonferroni post-hoc multiple comparisons in SPSS (IBM SPSS Statistics 24). These curves were then analysed using the incremental area under the curve (IAUC) function followed by a two-way ANOVA with the Bonferroni post-hoc analysis (GraphPad Prism 7 software). Differences between diet groups only were analysed using an unpaired *t*-test (GraphPad Prism 7 Software). Furthermore, *p*-values < 0.05 were considered statistically significant for all tests. Results are presented as mean ± standard error of the mean (SEM). The Cohen’s D (d) equation was used to examine the magnitude of the effect of the changes in gene expression. A large effect is considered when d > 0.8, a medium effect when d = 0.5–0.79 and a small effect when d = 0.2–0.49. 

## 3. Results

Although it is not possible to create an isocaloric diet with a 60% fat diet comparator, as the volume required to match this for the normal chow is too large, there was no significant difference in the volume of chow consumed between the two diet groups.

### 3.1. Body Weight

**Figure 1:**
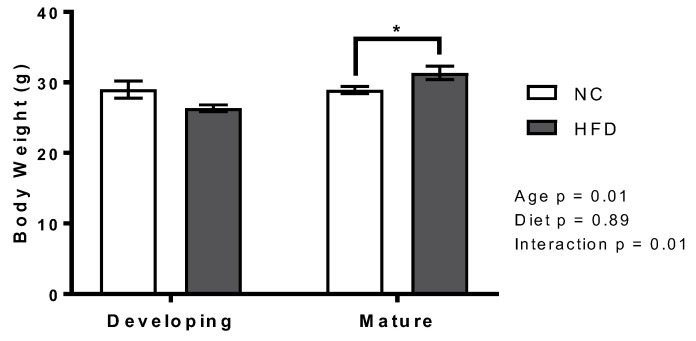
The high fat diet increased total body weight in the mature mice, but no change was seen between diets in the developing mice. Total body weight of the developing and mature mice after 10 weeks on normal chow (NC) and high fat diets (HFD) were measured by electronic scale. Results are presented as mean ± SEM (*n* = 7 per group), * represents *p* < 0.05 (2-way ANOVA with unpaired *t*-test to compare diets within age groups).

In developing mice, there was no statistical difference in the total body weight of NC mice (29 ± 1.22 g) and HFD mice (26.3 ± 0.5 g). Mature mice that were fed the HFD had a higher body weight than NC-fed mice (31.4 ± 0.89 g compared to 29 ± 0.52 g, respectively, *p* = 0.04; Figure 1). 

### 3.2. oGTT

**Figure 2:**
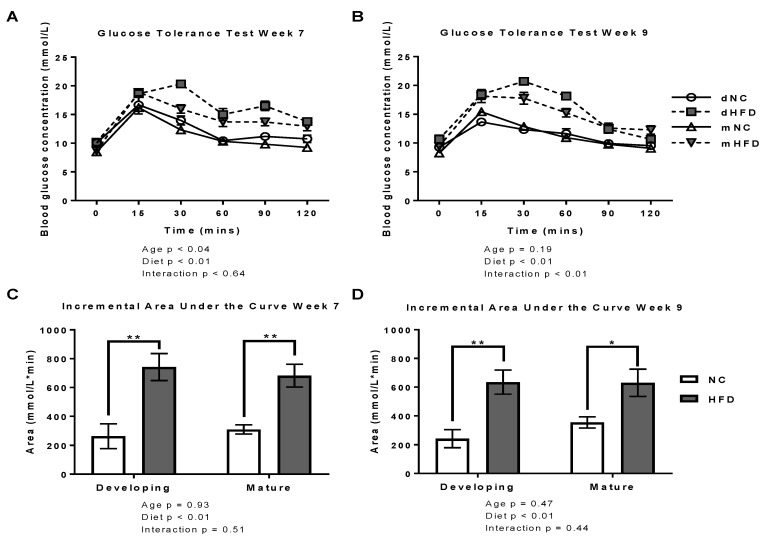
The high fat diet decreased glucose tolerance in both developing and mature mice. (**A**,**B**) Oral glucose tolerance test response curved during week 7 and 9 on diets for developing normal chow (dNC), developing high fat diet (dHFD), mature normal chow (mNC) and mature high fat diet (mHFD) groups. Mice received a 2 g/kg body weight dose of glucose following a 6 h fast. Results are presented as mean ± SEM (*n* = 7 per group), (general liner mixed model repeated measures analysis followed by Bonferroni post hoc multiple comparisons). (**C**,**D**) Corresponding incremental area under the curve analysed by the Graphpad prism function. Results are presented as mean ± SEM (*n* = 7 per group), * represents *p* < 0.05, ** represents *p* < 0.01, (2-way ANOVA followed by an unpaired t-test to compare diets within age groups).

At baseline and throughout the oGTT, mice fed the HFD from both age groups showed increased blood glucose levels by weeks 7 (*p* < 0.01) and 9 (*p* < 0.01) of the study (Figure 2A,B). The calculated iAUC for week 7 was higher for developing HFD (*p* < 0.01) and mature HFD (*p* < 0.01) compared to their NC controls (Figure 2C). This result was maintained at week 9 for developing (*p* < 0.01) and mature mice groups (*p* < 0.02; Figure 2D).

### 3.3. µ-CT 

**Figure 3:**
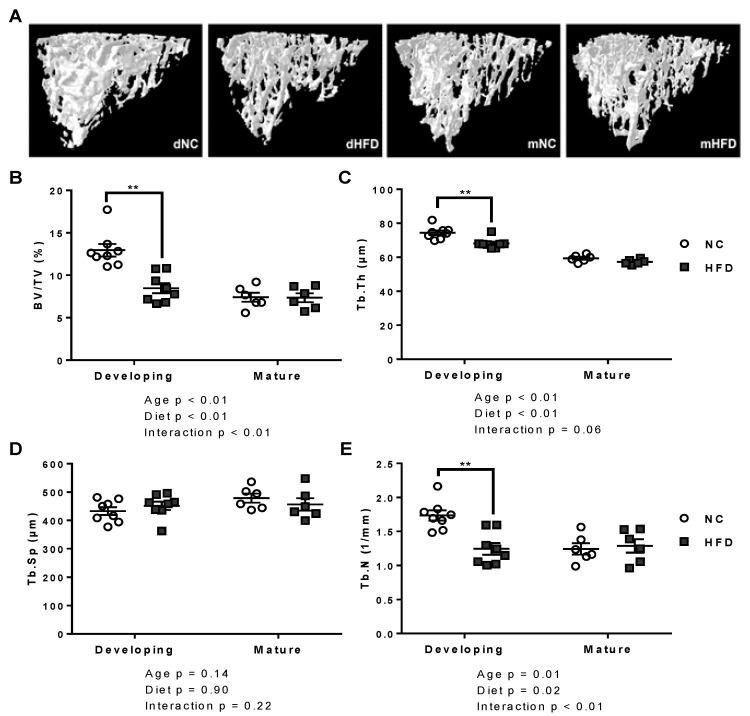
The high fat diet compromised tibial trabecular bone microarchitecture in the developing mice but resulted in no change within mature groups. (**A**) Ex vivo µCT volumes of interest for tibial trabecular bone analysis in developing normal chow (dNC), developing high fat diet (dHFD), mature normal chow (mNC) and mature high fat diet (mHFD) groups. (**B**–**E**) Bone volume to total volume ratio (BV/TV), trabecular thickness (Tb.Th), trabecular separation (Tb.Sp) and trabecular number (Tb.N) in developing and mature mice on normal chow (NC) and high fat diets (HFD). Results are presented as scatter plots with mean ± SEM (*n* = 7 per group), ** represents *p* < 0.01, (2-way ANOVA followed by Bonferroni post-hoc analysis comparing diet within age groups).

**Figure 4:**
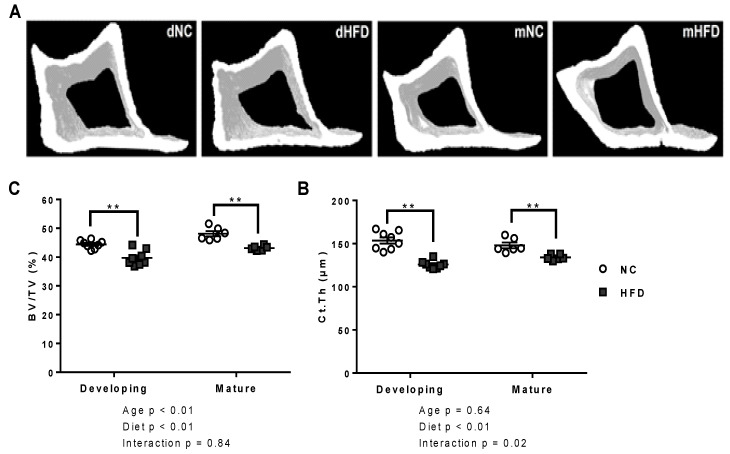
The high fat diet adversely affected tibial cortical bone outcomes in both developing and mature age groups. (**A**) Ex vivo µCT volumes of interest for tibial cortical bone analysis in developing normal chow (dNC), developing high fat diet (dHFD), mature normal chow (mNC) and mature high fat diet (mHFD) groups. (**B**,**C**) Bone volume to total volume ratio (BV/TV) and cortical thickness (Ct.Th) of developing and mature mice on normal chow (NC) and high fat diets (HFD). Results are presented as scatter plots with mean ± SEM (*n* = 7 per group), ** represents *p* < 0.01, (2-way ANOVA followed by Bonferroni post-hoc analysis comparing diet within age groups).

The developing HFD group showed extensive changes in the morphology of both trabecular and cortical bone compared to the developing NC controls (Table 1). Significant reductions in trabecular BV/TV, trabecular thickness (Tb.Th) and trabecular number (Tb.N) were observed (*p* < 0.01 for all), with no change in trabecular separation (Tb.Sp; Figure 3A–E). Cortical bone parameters were also significantly affected, with reduced cortical BV/TV ratio (*p* < 0.01) and thickness (Ct.Th; *p* < 0.01; Figure 4A–C). 

All trabecular parameters remained unchanged between diet groups in the mature age group (Figure 3A–E). The mature HFD mice showed significantly reduced cortical parameters, including cortical BV/TV (*p* < 0.01) and Ct.Th (*p* < 0.01), compared to the mature NC mice (Table 1 and Figure 4A–C). 

### 3.4. Bone Histomorphometry 

**Figure 5:**
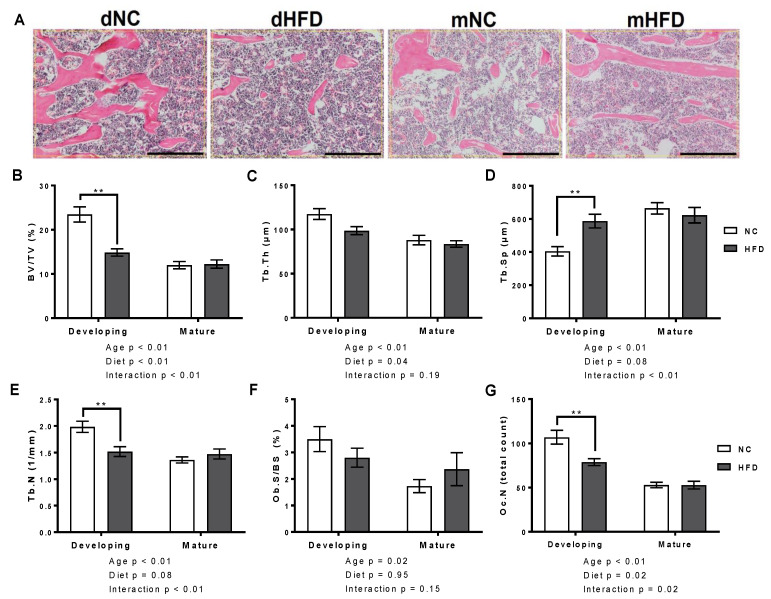
The high fat diet adversely affected structural and cellular histomorphometric outcomes in developing mice but had no effect in mature mice. (**A**) Ex vivo 2D histomorphometry regions of interest at 20x magnification analysed for developing normal chow (dNC), developing high fat diet (dHFD), mature normal chow (mNC) and mature high fat diet (mHFD) groups. Scale bar represents 1 mm. (**B**–**G**) Bone volume to total volume ratio (BV/TV), trabecular thickness (Tb.Th), trabecular spacing (Tb.Sp), trabecular number (Tb.N), osteoblast surface to bone surface ration (Ob.S/BS) and osteocyte count (Oc.N) of developing and mature mice on normal chow (NC) and high fat diets (HFD). Results are presented as mean ± SEM (*n* = 7 per group), ** represents *p* < 0.01, (2-way ANOVA followed by Bonferroni post-hoc analysis comparing diet within age groups).

Consistent with the µCT results, 2D histomorphometry revealed negative structural alterations in trabecular bone of the developing mice following HFD but no changes in the trabecular bone of the mature mice (Table 2 and Figure 5B–D). The developing HFD mice presented with decreased trabecular BV/TV, decreased Tb.N and increased Tb.Sp (*p* < 0.01 for all; Figure 5B,D,E). No significant effect was observed for Tb.Th (*p* = 0.07; Figure 5B). Upon analysis of the cellular properties, we observed the developing HFD group had a reduced osteocyte count (Oc.N; *p* < 0.01), relative to developing NC mice, while the osteoblast surface to bone surface ratio (Ob.S/BS) was not significantly affected (Figure 5F,G). Cellular properties for the mature mice remained unchanged between the two diet groups (Figure 5F,G). 

### 3.5. Biomechanical Properties (Three-Point Bending Tests)

Mechanical properties (maximal load and stiffness) were measured by three-point bending tests. Both age and diet had a significant effect on the maximal load and stiffness of the tibiae (2-way ANOVA *p <* 0.01 for all; Table 3). Consumption of the HFD resulted in significant reductions in the maximal load in both developing and mature mice (*p* < 0.01 for both), and there was a significant interaction effect with age (*p* < 0.01) for maximal load, with the developing HFD mice displaying greater reductions in maximal load than the older HFD when compared to age matched NC controls. The high fat diet increased stiffness in both age groups, from 60.5 N/mm in the developing NC group to 66.6 N/mm in the developing HFD group; and from 63.7 N/mm in the mature NC group to 71.8 N/mm in the mature HFD group (Table 3).

### 3.6. Gene Expression Analysis

**Figure 6:**
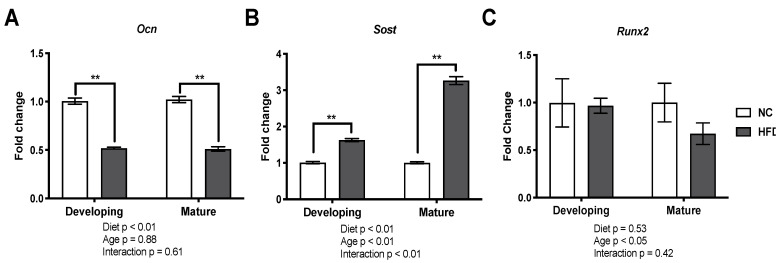
The high fat diet groups showed altered OCN and SOST gene expression in both the mature and developing mice. Isolated tibiae were flushed of bone marrow and the RNA was isolated, converted to cDNA, quantified by real time PCR and normalized to β-actin for (**A**) osteocalcin (*Ocn*), (**B**) sclerostin (*Sost*) and (**C**) runt-related transcription factor 2 (*Runx2*). Results are presented as mean fold change in expression ± SEM of normal chow (NC) and high fat diet (HFD) groups (*n* = 6 per group for OCN and SOST; *n* = 3 per group for Runx2; all individual samples run in triplicate), ** represents *p* < 0.01, (2-way ANOVA followed by Bonferroni post-hoc analysis for comparison within age groups).

The HFD reduced gene expression of *Ocn* by approximately half in both age groups (*p* < 0.01 for both; effect size: d = 9 for developing and d = 8.3 for mature; Figure 6A). The developing HFD group showed an increased relative expression of *Sost* (1.6 ± 0.04; *p* < 0.01; effect size: d = 7; Figure 6B). The mature HFD group also showed an increased relative expression; however, this was more marked in the developing mice as the expression increased to 3.27 ± 0.11 (*p* < 0.01; effect size: d = 13; Figure 6B). Runx2 was not significantly altered in either group (*p* > 0.05 for both) although the effect of the diet was significant (2-way ANOVA *p* < 0.05), and there was a large clinically relevant reduction in Runx2 in the mature group following the HFD as determined by Cohens’ d (d = 0.1 for developing and d = 1.15 for mature; Figure 6C).

### 3.7. Western Blot Analysis

**Figure 7:**
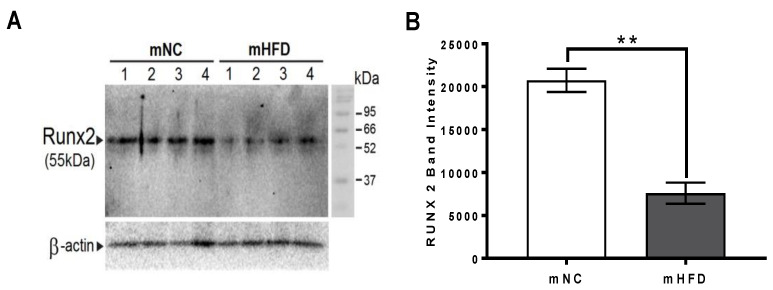
The high fat diet decreased runt-related transcription factor 2 (Runx2) protein expression in the femora of mature mice. (**A**) Western blotting of Runx2 protein expression in the right tibia flushed of bone marrow in mature normal chow (mNC) and mature high fat diet (mHFD) mice (*n* = 4 in each group). (**B**) Runx2 band intensity acquired using the BioRad ChemiDoc imaging system and band intensity quantified using ImageJ. Results are presented as mean ± SEM, ** represents *p* < 0.01, (unpaired *t*-test).

Runx2 protein were observed at the expected molecular weight, 55 kDA (Figure 7A). Mature HFD mice were found to have significantly reduced levels of Runx2 compared to the mature NC mice (*p* < 0.01; Figure 7B).

## 4. Discussion

In this study, we investigated the effects of the HFD on bone parameters in mice both before and after the acquisition of skeletal maturity. We report that in the developing mice, despite no change in body weight, glucose tolerance was reduced, and they had marked deficits in both trabecular and cortical bone microarchitecture as well as altered gene expression, suggesting decreased bone formation. The mice fed the HFD post-skeletal maturity presented with increased body weight, reduced glucose tolerance, compromised bone microarchitecture in the cortical bone and a gene and protein expression profile indicative of severely decreased bone formation. These findings suggest that the post-skeletal maturity model showed a more analogous phenotype to late-onset glucose intolerance and T2DM in humans. Furthermore, it is the superior animal model for investigations into the mechanisms of skeletal fragility in these diseases and future investigations of therapeutic interventions. In addition, we suggest that the HFD induces decreased osteoblastic activity via decreased OCN and increased SOST expression in both age groups, as well as decreased RUNX2 expression, particularly at the protein level in the mature mice.

While cases of non-obese T2DM are becoming increasingly prevalent, the majority of cases are obesity related and occur later in life [39]. To represent these cases, the current study aimed to produce a model applicable to late-onset, obesity related impaired glucose tolerance without interventions such as streptozotocin. The C57BL/6J model has been extensively investigated to study diet induced obesity and metabolic syndrome as it displays increased body weight/fat mass accumulation, leptin resistance and adipose distribution, closely mimicking the disease progression that occurs in humans [40,41]. While no analysis of body composition or fat mass is reported, it is well established in this model, and indeed, the HFD mature mice in this study showed increased body weight [41,42]. The developing mice fed the HFD did not show an increase in total body weight compared to normal chow. This is likely due to various physiological mechanisms, such as higher basal metabolic rates, that prevent an increase in body weight in younger adult mice despite being challenged by the diet. This is also supported by previous reports that indicate ageing mice have accelerated liver injury, insulin resistance, dyslipidaemia, fat storage and gut microbiota profile, following an HFD [43].

The observed responses to oGTT suggest metabolic abnormalities in glucose handling in these mice. However, an important consideration of the C57BL/6J diet induced model is that these mice do not develop frank diabetes [43]. An IAUC analysis of oGTT responses indicated impaired glucose tolerance in the HFD mice of both age groups, likely synergised with hyperlipidaemia, hyperinsulinemia and hyperleptinemia, all of which have been previously investigated in this model [41,42]. 

Previous studies analysing structural skeletal outcomes in C57BL/6J mice fed the HFD have primarily initiated diets before skeletal maturity [44,45,46]. In the C57BL/6J model, maturity has been defined between 12 and 42 weeks of age [34]. In order to represent the vast majority of impaired glucose tolerance/T2DM cases that occur later in life [39], we aimed to identify a model that began to show diabetic-like characteristics post-skeletal maturity. These data support previous findings that the HFD has a negative effect on skeletal microarchitecture [44,45,46]. However, we report the novel findings that this effect is more apparent when the diet is commenced during an age of development, even if at a young adult stage (8 week old mice). Mature HFD mice not only demonstrated less drastic changes in cortical bone outcomes but showed no apparent change in trabecular bone outcomes. Similar results have been found in a previous study showing that bone microarchitecture of younger mice was more susceptible to the adverse effects of the HFD [47]. The studies differ, however, in that the adult mice of the previous study still showed an effect on trabecular outcomes in adult mice when starting the diet at 20 weeks of age [47]. The skeletal changes seen in the mature mice of this study reflected structural changes seen in obese and T2DM patients who present with altered cortical bone microarchitecture [28,31], but not trabecular bone [14,15,26,27]. As the developing mice over represent changes in microarchitecture, we propose the use of mature mice to further characterize mechanisms of skeletal fragility, such as the introduction of AGE products into the bone matrix and reduced bone turnover, by also investigating the activity of osteoclasts for late-onset patients with impaired glucose tolerance and/or T2DM. A consideration when comparing the diet responses between these two age groups are that both diets in the mature mice showed similar microarchitectural properties as those in the developing HFD group. This could be due to either normal variation between litters, or that the C57BL/6J model is known to show age related bone loss from 6 weeks of age [48]. 

More importantly, while microarchitectural changes were only observed in the developing group and either undetected or modest, at most, in the mature group, skeletal fragility was confirmed by strength testing that verified that the HFD bones of both age groups are indeed more fragile, with greater stiffness (indicating brittleness) and lower maximal load required to fracture. In conjunction with only small changes in microarchitecture, these results indicate a compromise in bone quality is the likely driving mechanism behind the increased likelihood of a fracture, rather than compromised architectural structure of the bones. Indeed, this is observed in humans in whom BMD is reported in the normal to high ranges [7,8,9,10], while fracture risk is up to 3-fold higher than in the healthy population [9,10,11].

Furthermore, 2D histomorphometry assessed structural properties in the trabecular region reflecting similar findings to µCT outcomes, and provided some insight into cell populations. We report that the HFD caused trabecular structural alterations in the developing mice but did not result in changes in the mature mice. In regard to cellular populations, we hypothesized a decrease in Ob.S/BS in the HFD mice; however, no differences between diet groups at either age were evident. This may be explained by the fact that the Ob.S/BS does not necessarily reflect the maturity or activity of these osteoblasts. Oc.N was decreased in the developing mice, although this is likely reflecting the decreased bone volume, as osteocytes reside within the bone matrix. 

Changes in gene and protein expression within the tibiae indicate that reduced osteoblast activity and function is a strong candidate for one potential mechanism for decreased bone quality and therefore increased skeletal fragility in T2DM. Decreased *Ocn* expression was apparent in the HFD mice of both age groups signifying decreased bone formation. Osteocalcin is secreted by osteoblasts and its expression is directly related to osteoblast differentiation and mineralization [49]. The decrease in *Ocn* expression indicates that while Ob.S/BS was unaffected in the HFD mice, the osteoblasts may not be fully differentiated or they remained quiescent. Both HFD age groups demonstrated increased *Sost* gene expression, with a 3-fold greater increase observed in the mature HFD mice. Sclerostin, secreted by osteocytes, is a Wnt pathway antagonist [50], and its expression is inversely correlated to bone formation, further supporting decreased bone formation. *Runx2* is considered the master regulator for osteoblast gene expression [51]. We quantified *Runx2* mRNA expression. Due to small bone sizes in the developing HFD group, we had a limited number of samples, which likely explains why results for *Runx2* mRNA expression showed large variation and no significant changes at either age. Thus, to further investigate if there were any changes, Runx2 protein levels were also investigated in the bones of the mature mice groups (again, the small bones from the developing HFD group rendered protein extraction difficult). We observed a significant decrease in Runx2 protein levels in the mature HFD mice, further indicating decreased osteoblast activity in these mice. 

The specific types of fats included in the diets we provided the mice in the current investigation are detailed in the methods above. Of course, not all fats are created equal. The distinction between the effects of saturated fats, and fats that contain antioxidants and bioactive food compounds such as polyphenols, is an important one to make when determining the effects of dietary fats on bone. Indeed, olive oil has been shown to support the proliferation and activity of osteoblasts [52]. Furthermore, diets that contain moderate levels of olive oil and nuts, such as the Mediterranean diet and a prudent (healthy) Western diet, have been shown to be more bone protective compared to an unhealthy Western diet that contains high levels of saturated fats; although, not all the benefits of such a diet come solely from the fats and oils [53,54].

Previous findings support a link between the hyperglycaemic, hyperinsulinemic and inflammatory environments in metabolic diseases such as glucose intolerance and T2DM with impaired bone remodelling. This has been comprehensively reviewed recently in Shahen et al., 2020 [55]. Thus, one limitation of our current study is that we have not investigated changes in inflammatory markers and direct links with the compromised bone quality we observed in our high fat fed mice. Future studies are warranted to determine the role of inflammation in the adverse effects of the HFD in this or similar models of dysmetabolism.

Similarly, studies from humans indicate a down-regulation of bone resorption in addition to bone formation in patients with T2DM [13,15,16]; thus, future studies in animals could also investigate the effects of a high fat diet on the activity of the osteoclasts, although it is again worth pointing out that our model is closer to generalized dysmetabolism and glucose intolerance than frank diabetes.

## 5. Conclusions

In conclusion, a skeletally mature HFD mouse model more accurately represents late-onset impaired glucose tolerance/T2DM cases in humans than the HFD mice prior to skeletal maturity, even at a young adult stage. The skeletally mature HFD model showed only minor changes in bone microarchitecture, including modest decreases in cortical thickness and bone volume. While structural changes were only minor, the mature HFD mice still exhibited increased stiffness and reductions in maximum load required for fracture (failure), indicating decreased bone quality. Changes in the gene and protein expression profiles indicate a decrease in osteoblast activity and likely reduced bone formation as one possible mechanism of skeletal fragility in these cases.

## Figures and Tables

**Table 1:** µCT analysis of tibial trabecular and cortical bone parameters in normal chow and high fat diet mice of developing and mature age groups.

		Developing		Mature	
		NC	HFD	NC	HFD
Trabecular	BV/TV (%)	12.95 ± 0.70	8.48 ± 0.55 **	7.40 ± 0.48	7.35 ± 0.48
	Tb.Th (µm)	74.46 ± 1.32	68.10 ± 1.08 **	59.41 ± 0.85	57.3 ± 0.61
	Tb.Sp (µm)	432.79 ± 13.75	451.34 ± 14.74	478.74 ± 15.79	456.21 ± 21.83
	Tb.N (1/mm)	1.73 ± 0.08	1.24 ± 0.08 **	1.24 ± 0.08	1.29 ± 0.10
Cortical	BV/TV (%)	44.41 ± 0.5	39.72 ± 0.93 **	48.13 ± 0.91	43.13 ± 0.33 **
	Ct.Th. (µm)	153.66 ± 3.67	125.70 ± 1.61 **	147.90 ± 3.33	134.08 ± 1.36 **

NC = normal chow; HFD = high fat diet; BV/TV = bone volume/total volume; Tb.Th = trabecular thickness; Tb.Sp = trabecular separation; Tb.N = trabecular number; Ct.Th = cortical thickness. Data are presented as the mean ± SEM (*n* = 6–8 per group), ** represents *p* < 0.01, (2-way ANOVA followed by Bonferroni post-hoc analysis comparing diet within age groups).

**Table 2:** The 2D histomorphometry of trabecular bone in normal chow and high fat diet mice of developing and mature age groups.

		Developing		Mature	
		NC	HFD	NC	HFD
Structural	BV/TV (%)	23.46 ± 1.71	14.85 ± 0.82 **	11.98 ± 0.83	12.22 ± 0.94
	Tb.Th (µm)	114.40 ± 6.11	98.60 ± 4.57	87.98 ± 5.37	83.48 ± 3.72
	Tb.Sp (µm)	404.70 ± 28.48	586.93 ± 41.43 **	664.19 ± 34.24	622.33 ± 46.92
	Tb.N (1/mm)	1.98 ± 0.11	1.52 ± 0.09 **	1.36 ± 0.06	1.47 ± 0.09
Cellular	Ob.S/BS (%)	3.50 ± 0.47	2.80 ± 0.36	1.73 ± 0.25	2.37 ± 0.62
	Oc.N	106.84 ± 7.70	78.78 ± 3.90 **	52.96 ± 3.16	52.83 ± 4.38

NC = normal chow; HFD = high fat diet; BV/TV = bone volume/total volume; Tb.Th = trabecular thickness; Tb.Sp = trabecular separation; Tb.N = trabecular number; Ob.S/BS = osteoblast surface/bone surface; Oc.N = osteocyte number. Data are presented as the mean ± SEM (*n* = 6–8 per group), ** represents *p* < 0.01, (2-way ANOVA followed by Bonferroni post-hoc analysis comparing diet within age groups).

**Table 3:** Biomechanical parameters obtained from a 3-point bending test of the femur.

		Developing		Mature	
		NC	HFD	NC	HFD
Mechanical	Max Load (N)	11.95 ± 0.01	8.64 ± 0.01 **	9.91 ± 0.02	8.66 ± 0.34 **
	Stiffness (N/mm)	60.53 ± 1.54	66.65 ± 0.27 *	63.75 ± 1.15	71.78 ± 0.83 **

NC = normal chow; HFD = high fat diet. Data presented as the mean ± SD (*n* = 3 per group: pilot data) ** *p* < 0.01, * *p* < 0.05 (2-way ANOVA followed by Bonferroni post-hoc analysis comparing diet within age groups).
